# Efficacy of a small molecule inhibitor of the transcriptional cofactor PC4 in prevention and treatment of non-small cell lung cancer

**DOI:** 10.1371/journal.pone.0230670

**Published:** 2020-03-31

**Authors:** Yan Zhang, Andrei Pavlov, Sohail Malik, Hong Chen, Nancy Kim, Ziqing Li, Xiaohong Zhang, Melvin L. DePamphilis, Robert G. Roeder, Hui Ge

**Affiliations:** 1 AscentGene, Inc., Gaithersburg, MD, United States of America; 2 Laboratory of Biochemistry and Molecular Biology, The Rockefeller University, New York, NY, United States of America; 3 National Institute of Child Health and Human Development, National Institutes of Health, Bethesda, MD, United States of America; H. Lee Moffitt Cancer Center & Research Institute, UNITED STATES

## Abstract

The human positive coactivator 4 (PC4) was originally identified as a multi-functional cofactor capable of mediating transcription activation by diverse gene- and tissue-specific activators. Recent studies suggest that PC4 might also function as a novel cancer biomarker and therapeutic target for different types of cancers. siRNA knockdown studies indicated that down-regulation of PC4 expression could inhibit tumorigeneicity of A549 non-small cell lung cancer tumor model in nude mice. Here we show that AG-1031, a small molecule identified by high throughput screening, can inhibit the double-stranded DNA binding activity of PC4, more effectively than its single-stranded DNA binding activity. AG-1031 also specifically inhibited PC4-dependent transcriptional activation in vitro using purified transcription factors. AG-1031 inhibited proliferation of several cultured cell lines derived from non-small cell lung cancers (NSCLC) and growth of tumors that formed from A549 cell xenografts in immuno-compromised mice. Moreover, pre-injection of AG-1031 in these mice not only reduced tumor size, but also prevented tumor formation in 20% of the animals. AG-1031 treated A549 cells and tumors from AG-1031 treated animals showed a significant decrease in the levels of both PC4 and VEGFC, a key mediator of angiogenesis in cancer. On the other hand, all tested mice remained constant weight during animal trials. These results demonstrated that AG-1031 could be a potential therapy for PC4-positive NSCLC.

## Introduction

In the United States, lung cancer is the leading cause of cancer-related deaths with 234,030 estimated new cases accounting for 13.5% of all new cancer cases and 154,050 deaths accounting for 25.3% of all cancer deaths in 2018 [1]. Non-small cell lung cancer (NSCLC) accounts for about 80% of all lung cancers and about 40% of NSCLC are often in an advanced stage when diagnosed. As a promising approach for NSCLC treatment, development of therapeutic agents that target specific molecular aberrations, including gene mutations, rearrangements, and other changes that cause DNA sequence alterations, have been advanced. Several targeted agents have been explored recently, including the antagonists of epidermal growth factor receptor 6 (EGFR6) and vascular endothelial growth factor receptor (VEGFR), and inhibitors of anaplastic lymphoma kinase (ALK). However, despite their initial promise in cancer chemotherapy, the use of current anti-angiogenic drugs, including bevacizumab (anti-VEGF-A), endostatin (bFGF inhibitor) and crizotinib (ALK and ROS1 inhibitor), had very little impact on the treatment of NSCLC [2–4]. Most recently, two monoclonal antibody-based drugs (nivolumab and pembrolizumab) that specifically target programmed cell death 1 (PD-1) were approved by the Food and Drug Adminstration for the treatment of advanced NSCLC following the first line therapy. Nevertheless, overall response rates to both agents were low [2], indicating that PD1- positive patients belong to a small group within the NSCLC population. The absence of targetable mutations in approximately 50% of NSCLC cases underscores the importance for developing alternative therapeutics for NSCLC treatment.

Although it has long been suspected that gene aberrations might only be part of the cause of cancer-related signaling pathways, altered expression of epigenetic factors may represent a major fundamental regulatory strategy involved in cancer-related process. Thus, alternative therapeutic strategies targeting novel epigenetic biomarkers may provide a significant breakthrough in treating cancer [4, 5].

Transcriptional positive cofactor 4 (PC4 in humans; Sub1 in yeast) was initially identified as a general cofactor mediating transcriptional activation of eukaryotic genes in vitro [6–9]. Apart from its roles in transcription by interacting with general transcription machinery and gene- or tissue-specific activators, PC4 also plays multiple cellular functions in vitro and in vivo. These include DNA-dependent processes, such as DNA replication by directly interacting with replication protein A complex (RPA), DNA repair by directly interacting with the XPG, and heterochromatinization by interacting with heterochromatin proteins. Thus, PC4 also plays essential roles in maintaining a dynamic chromatin state and in heterochromatin gene silencing [10–12]. The activity of PC4 is negatively regulated by phosphorylation of several kinases, particularly, casein kinase II [13, 14]. Although PC4 binds both single- and double-stranded DNA in a sequence-independent manner, acetylation of PC4 by p300 enhances its ability to bind dsDNA [15, 16].

Correlation of PC4 expression and tumor progression has been observed in different tumor types including astrocytoma [17], lung carcinoma [18], small cell lung cancer [19] and esophageal squamous cell carcinoma [20]. Overexpression of PC4 in a population of normal dermal multipotent fibroblast cells resulted in a tumorigenic transformation of the cells, indicating the role of PC4 in tumor development and progression. [18]. Large-scale integration of cancer microarray data further validated that PC4 is one of 46 genes in the cancer signature among 21 major human cancer types [21]. Knockdown of PC4 expression by sequence-specific small interfering RNA in human NSCLC cells and in pre-established NSCLC cell xenografts in mice showed significant inhibition on growth of tumor cells and tumor, respectively, suggesting that PC4, a validated novel cancer biomarker, could be a potential therapeutic target for the treatment of NSCLC [18, 22].

Because the double-stranded DNA binding activity is correlated directly to the coactivation activity of PC4, using an established double-stranded DNA binding platform, we were able to identify a small molecule, AG-1031, that inhibited binding of PC4 to dsDNA, and, less effectively, binding to ssDNA. An *in vitro* transcription assay showed that AG-1031 specifically inhibited PC4-dependent transcription in a concentration-dependent manner. In addition to inhibiting proliferation of different human NSCLC cells, injection of AG-1031 into mice bearing an NSCLC cell (A549) xenograft resulted in an inhibition of tumor growth. Furthermore, pre-injection of AG-1031 could prevent tumor formation in xenograft mice. Thus, AG-1031 could be the first small molecule that specifically targets the transcription cofactor PC4 and a paradigm for anti-cancer drugs that target transcriptional cofactors.

## Materials and methods

### Screening of small molecules that specifically inhibit the double-stranded DNA-binding activity of PC4

Recombinant PC4 was expressed in *E*. *coli* and purified by conventional chromatography methods to over 95% homogeneity [6]. Nun-Immuno MaxiSorp plates were coated with 1 μM PC4 in a buffer containing 20 mM HEPES-Na (pH 7.9), 10% glycerol, 0.2 mM EDTA, and 0.1 M KCl (50 μl/well) for 1h at room temperature, washed 3 times with 100 μl 1xPBS supplemented with 0.05% Tween 20, and blocked with 100 μl of 1% BSA in this buffer for 1h at room temperature. All chemicals were diluted with the same buffer to a concentration of 3 mM for use in the screen assay. After washing, the plate wells 3 times with 1xPBS/0.05% Tween 20, 25 μl of the chemical solutions were applied to each well, followed by addition of 25 μl of 50 nM biotinylated dsDNA. After incubating for 1h at room temperature and washing the wells 3 times with the same buffer, 50 μl /well of 1:5000 HRP- conjugated Streptavidin in 1xPBS containing 1% BSA were applied to the plates. After 1h incubation at room temperature, the plate wells were washed twice with 100 μl and once with 200 μl of 1xPBS/0.05% Tween 20, TMB substrate was added (50 μl /well). The reaction was stopped with 50 μl/well 20% phosphoric acid after 5 min incubation and the dsDNA-binding activity of PC4 was then monitored by measuring the absorbance at 450 nm in a microplate reader. Reactions in triplicate were repeated twice with independent dilutions of 10 mg/ml stock solutions in DMSO. For inhibitors the concentration curves were measured, in which the effects were consistent with the inhibition in the screening reactions.

### *In vitro* transcription

*In vitro* transcription reactions in the presence of ^32^P-CTP from an HNF-4-driven G-free DNA template under the control of the adenovirus major late core promoter were reconstituted with purified factors essentially as described previously [23]. Briefly, reactions contained affinity purified RNA polymerase II, TFIID, and TFIIH and recombinant TFIIA, TFIIB, TFIIE, TFIIF, and the HNF-4 activator in the presence of absence of recombinant PC4 (bacterially-expressed and purified, 150 ng). Increasing amounts (0-200μM) of AG-1031 (W&J ChemPharm, Cat# 100723–31, Lot# wj100126) were added, as indicated. After incubation at 30°C for 60 min, reactions were stopped and the ^32^P labeled transcripts were analyzed by gel electrophoresis and visualized by autoradiography. The density of individual bands was quantified using VisionWorks LS Image Acquisition and Analysis Software.

### Cell culture and cell proliferation assay

Human lung adenocarcinoma cell lines A549, H1299, H841 and H460 were obtained from ATCC. A549 cells were grown in DMEM/F12 medium. H1299, H841, and H460 cells were grown in RPMI medium (Sigma). All cell culture media contained 10% FBS and 100 units/ml of Penicillin-Streptomycin. All cells were incubated in a 37°C/5%CO_2_ humidified incubator. One day before treatment with a chemical compound, each of the four cell lines, A549, H1299, H841, and H460, were seeded into 96-well plates at 10,000 cells/well (100 μl) in triplicate. On the day of treatment, the culture medium was removed, and cells were treated with different concentrations of AG-1031 in fresh culture medium. Following an additional incubation of 48h at 37°C/5%CO_2_, cell proliferation assays was performed with Cell Counting Kit-8 (Dojindo Laboratories, #CK04), by adding 10 μl of reagent to each well with 100 μl of fresh culture medium. After incubating for an additional 2 h to 3 h at 37°C/5%CO_2_, OD_450nm_ was measured in a 96-well plate reader (Victor, 1420 Multilabel counter). Data analyses were performed with Excel and plotted as relative mean OD_450nm_ +/- standard error of the mean.

### Animal studies

Animals were stayed in the NIH-6B animal facility and kept in an environment with controlled temperature, humidity and a light/dark cycle of 12hrs, under veterinary surveillance for animal health and comfort. Four mice were kept in each cage with sterilized food, drinking water, and bedding. Cages were changed twice a week. The study was reviewed and approved by the NIH-NICHD Animal Care and Use Committee (ACUC). The protocol of the animal care and experimental design were approved by the National Institutes of Health, United States (Protocol number ASP 11–056). All injections (subcutaneous and intraperitoneally) were done under isoflurane anesthesia with anesthesia machine to minimize animal suffering and distress and to eliminate the extemporaneous injury to the mice. The mouse was euthanized either when the tumor reaches a maximum diameter of 2cm, or when it begins to ulcerate. Any mouse that was lunched, lethargic, dehydrated, or loss of >20% body weight was euthanized. At the end of trial, mice were euthanized using anesthetic overdose following by decapitation. SHO mice (Crl:SHO-*Prkdc_scid_Hr_hr_*/6-8weeks) were purchased from Charles River. Data resulted from animal studies were evaluated statistically using GraphPad Prism 6.0 software.

**I. Treatment:** A549 cells were injected subcutaneously on the right flank of 8 week-old mice (3x10^6^ cells/mouse). Tumors appeared around one week post-inoculation. When the tumor size reached 90 mm3 (around two weeks post-inoculation), a total of 14 mice were randomly divided into two groups (Treatment and Vehicle). Each mouse in the Treatment Group was injected intraperitoneally (IP) with AG-1031 (100 μl of 0.4 μg/μl; 40 μg/mouse) every other day for 28 days. Each mouse in the Vehicle Group received the same volume of H_2_O. Tumor size was measured using sliding calipers and calculated using the following formula: mm3 = L/2 x W/2 x H/2 x 4/3 x 3.14159

The resulting data were plotted as relative mean tumor volume +/- standard error of the mean.

**II. Prevention of tumor development:** A total of 10 mice were divided randomly into two groups (Vaccine & Control). The mice in the Vaccine Group were injected IP with 100 μl of AG-1031 at 0.4 μg/μl for a total 40 μg/mouse, once a week for 3 weeks before mice were inoculated with tumor cells to produce a xenograft. A549 cells (3x10^6^) were then injected subcutaneously on the right flank of each mouse. Tumors appeared around one week post-inoculation. Tumor size was measured as above.

### Immunohistochemistry

Mice (from treatment setting) were sacrificed when the tumor xenografts were at the stage of day-28 (day 1 was defined as the day of the first AG-1031 injection). Tumors were dissected and tumor tissues were fixed by placing them in formalin overnight. After washing three times with PBS, the fixed tumor tissue was then prepared for immunohistochemistry. The tumor slides were permeabilized with 0.3% Triton X100 in PBS for 5 min. After blocking non-specific staining by incubating the tissue for 30 min at room temperature in 3% bovine serum albumin, the tumor tissue slides were stained either with mouse monoclonal anti- PC4 antibody (AscentGene, USA, 1:1000 dilution) or with rabbit polyclonal anti-VEGFC (Vascular Endothelial Growth Factor C) antibody (Abcam, Cat# ab135506, USA, 1:500 dilution) at 4°C overnight. The slides were then washed three times with PBST and mounted with mounting medium containing either goat anti-mouse IgG conjugated with Rhodamine for PC4 (Pierce/1:2000 dilution), or goat anti-rabbit IgG antibody, Alexa Fluor 568 for VEGFC (TheromFisher1:2000 dilution), both of which contained DAPI to stain nuclear DNA (Vector Labs, Inc. Cat# H-1200).

### Western blotting analysis

To detect the effect of AG-1031 on the expression of PC4, A549 cells were treated with 0.3μM, 1μM and 3μM of AG1031 for 72 hours and then the whole cell lysates were prepared. 5μg of total proteins from each sample were loaded in each well and detected using the polyclonal antibody against PC4 (AscentGene). β-actin was used as an internal control detected by the monoclonal antibody against β-actin (Genscript).

To detect the effect of AG-1031 on the expression of PC4 of drug-treated animals, mice were sacrificed when the tumor xenografts were at the day 28 stage (day 1 was defined as the day of the first AG-1031 injection). Tumors were dissected and tumor tissues were stored at -80°C. Total proteins were extracted from the frozen tumor tissues with RIPA Buffer (50mM Tris-Cl, pH7.9, 150mM NaCl, 0.1% SDS, 0.5% Na-deoxycholate and 1% NP-40). Protein concentration was determined using the Bio-Rad protein assay dye reagent kit (Bio-Rad, Cat#500–0006). 50 μg of tumor tissue extract from each animal were analyzed by electrophoresis in 4–12% SDS-PAGE (Invitrogen, NP0323 BOX), transferred to 0.2 μm pore size nitrocellulose membrane and incubated with the polyclonal antibody against PC4 and the polyclonal antibody against TBP (TATA-binding protein, AscentGene) as an internal control. HRP-conjugated secondary antibody was then used for the detection of specific antigen.

## Results and discussion

### Identification of PC4 inhibitor AG-1031

**Fig 1:**
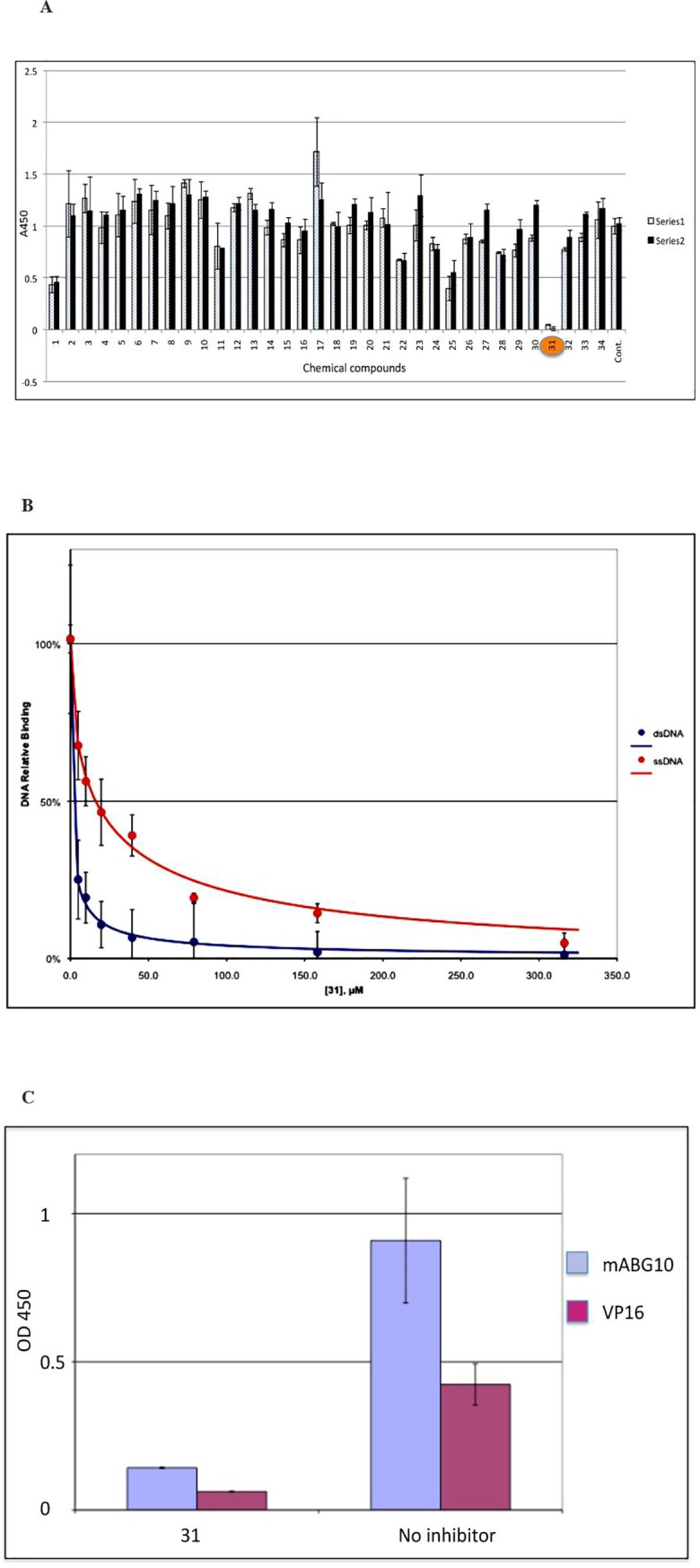
A) Identification of PC4 inhibitor AG-1031. An in vitro screening assay was developed based on the dsDNA binding activity of PC4. Reactions (in triplicate) were repeated twice with independent dilutions from the stock solutions of 34 active pharmaceutical ingredients (APIs) for non-oncology indications. The control reaction (Cont) contains no compound and represents the binding activity of PC4 to the dsDNA, which is normalized as the value “1”. AG-1031 (#31, highlighted) was identified as the most effective PC4 inhibitor. B) Interference of AG-1031 on PC4 binding to ds- or ssDNA. AG-1031 (#31) was identified as the most effective PC4 inhibitor by using an in vitro screen assay based on the dsDNA binding activity of PC4. The 50% inhibitory concentration (IC_50_) of AG-1031 for dsDNA binding was 2.5 μM, and 15 μM for ssDNA binding, suggesting that AG-1031 preferentially inhibited binding of PC4 to dsDNA rather than to ssDNA. C) AG-1031 inhibits PC4 binding to monoclonal antibody and activator VP16. Recombinant PC4 was immobilized on a 96-well plate and incubated with either the monoclonal antibody against PC4 (mABG10) or recombinant 6His-tagged VP16 protein in the presence (#31) or absence (No inhibitor) of AG-1031. Bound monoclonal antibody was detected with anti-mouse HRP, and 6His-tagged VP16 was detected with anti-6His mouse mAb followed by anti-mouse HRP.

Since PC4 is a nuclear protein that binds nucleosomes and affects chromatin structure, only small molecules have the potential to rapidly diffuse across cell and nuclear membranes. These small molecules are then capable of targeting PC4 and down-regulating the expression of PC4-mediated oncogenes. Based on the correlation of dsDNA binding activity and its transcription activity, we developed an ELISA–based assay system, in which purified recombinant PC4 was coated on a 96-well plate and then incubated with biotinylated dsDNA oligos. PC4-bound biotinylated oligos were then detected by using HRP-conjugated streptavidin. We screened >1000 drug-like compounds including 86 active pharmaceutical ingredients (API) for oncology indication (NCI DTP Approved Oncology Drugs Set- 2010) and 34 for non-oncology indication. Only AG-1031, one of the 34 APIs for non-oncology indication, was identified (Fig 1A, compound #31). The 50% inhibitory concentration (IC50) of AG-1031 was 2.5 μM for dsDNA binding, and 15 μM for ssDNA binding (Fig 1B), revealing that AG-1031 preferentially inhibited binding of PC4 to dsDNA rather than to ssDNA. AG-1031 also interfered with PC4 binding to a monoclonal antibody against PC4 and with PC4 binding to the transcription activator VP16 (Fig 1C), suggesting that AG-1031 bound to a site on PC4 that is essential for binding dsDNA, VP16 and the monoclonal antibody.

### AG-1031 inhibits PC4-dependent transcription in vitro

**Fig 2:**
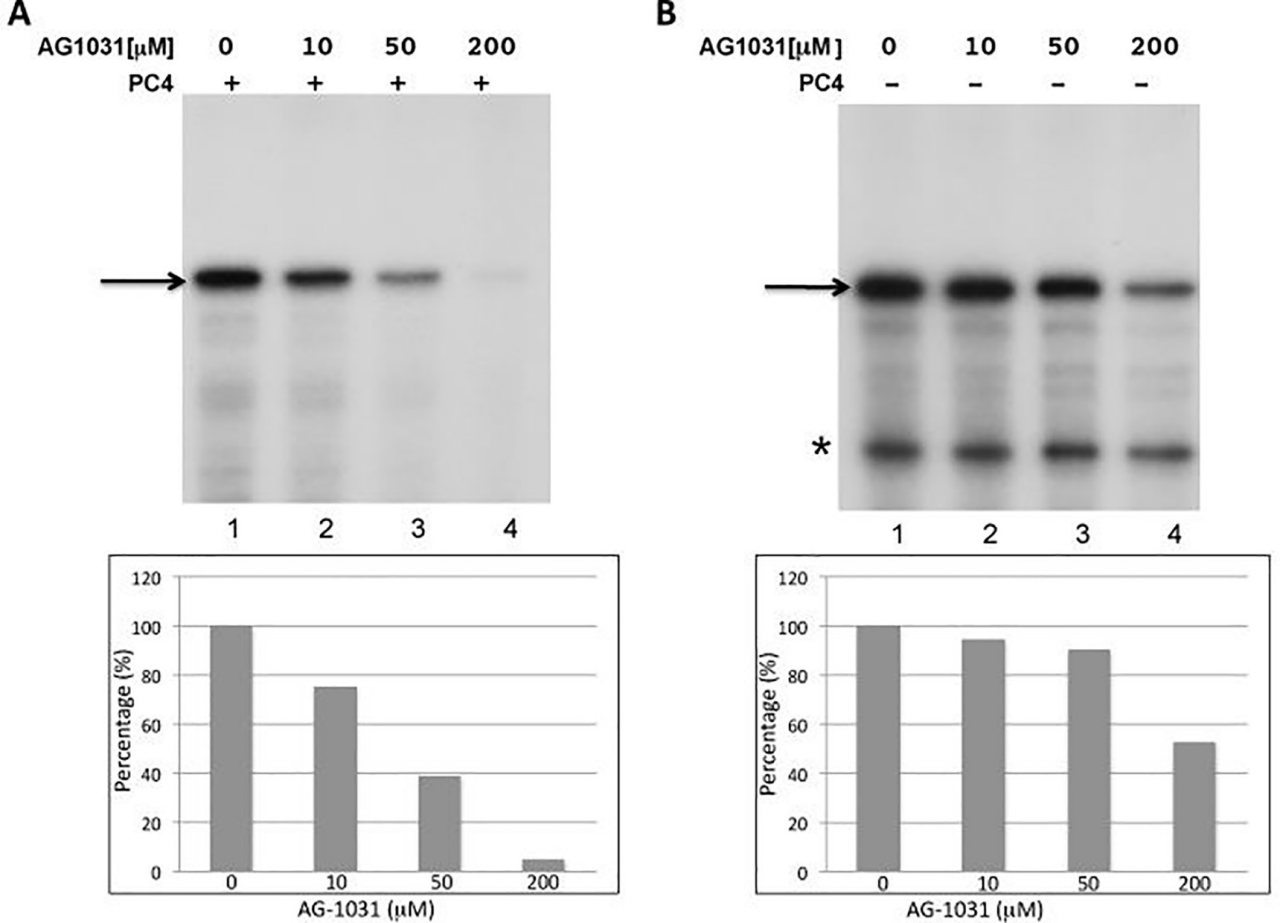
In vitro transcriptions contained affinity purified RNA polymerase II, TFIID, and TFIIH and recombinant TFIIA, TFIIB, TFIIE, TFIIF, and the HNF-4 activator in the presence (A) or absence (B) of recombinant PC4. Drug additions were as indicated. Arrows identify the full-length G- free product originating at the authentic transcription start site. The asterisk in panel B identifies a non- specific RNA that is observed in the absence of PC4. Specific products were quantified and percent inhibition was plotted as a function of AG-1031 concentration (lower panels).

AG-1031 is a small molecule that was approved by the U.S. Food and Drug Administration for non-oncology indication. To confirm that AG-1031 can inhibit PC4-mediated transcription, the compound was titrated into in vitro transcription reactions reconstituted with purified transcription factors, the activator HNF-4, and a cognate DNA template (Fig 2). As expected, AG-1031 inhibited PC4-dependent transcription in a concentration-dependent manner (Fig 2A), By contrast, only minor inhibition was observed at concentrations up to 50 μM when reactions were carried out in the absence of PC4 (Fig 2B). Note that when PC4 is not present, the transcription system yields an additional non-specific band, which could be resulting from a cryptic start site or represent a prematurely terminated product. Interestingly, this transcript (asterisk) is much less sensitive to AG-1031 compared to the full-length authentic transcript.

### AG-1031 induces loss of viability in NSCLC cells

**Fig 3:**
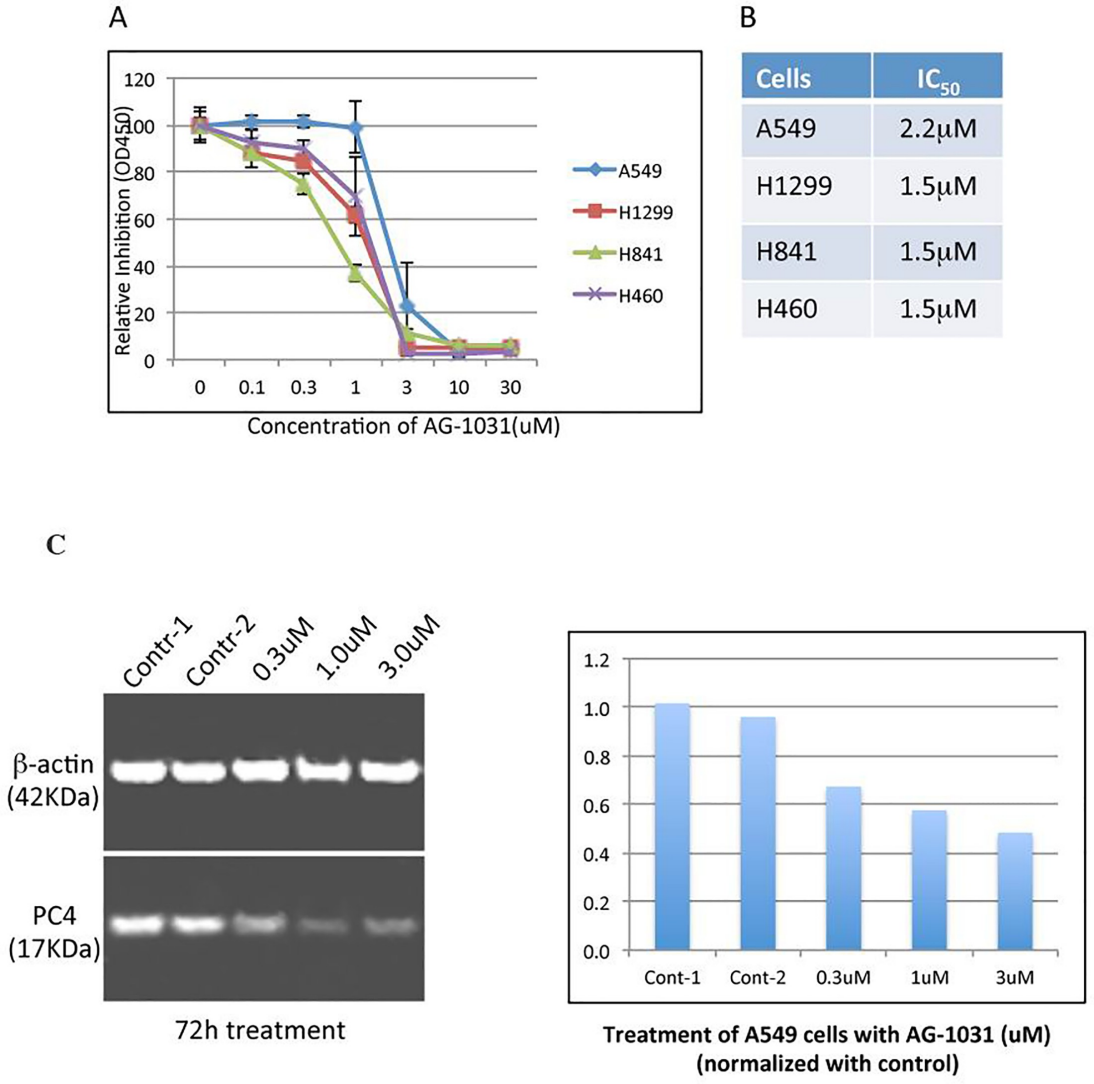
AG-1031 inhibits the viability of lung adenocarcinoma cell lines. NSCLC cell lines A549, H1299, H841 and H460 were incubated in the presence of increasing concentrations of AG-1031. Viable cells were counted 48 hrs post-treatment by using a cell counting kit and measured using a 96- well plate reader at OD_450nm_. AG-1031 showed inhibitory effect on all of four cell lines with IC50 between 1–2 μM (Fig 3A and 3B). Western blot analysis showed that treatment of A549 cells with AG-1031 resulted in down-regulation of PC4 in a concentration-dependent manner (Fig 3C/left panel). Expression of β-actin was used as the control. The density of PC4 expression was semi-quantified and the relative inhibition was plotted (Fig 3C/right panel).

It was reported that down-regulation of PC4 by PC4-specific siRNA could inhibit proliferation of different human NSCLC cell lines [18]. To assess if the PC4 inhibitor AG-1031 could also inhibit proliferation of NSCLC cells, human NSCLC cell lines A549, H1299, H841 and H460 were cultured in the presence of different concentrations of AG-1031 and the viability of individual cell lines was monitored using a cell viability (cell proliferation) assay at 48 hrs post-treatment. The result in Fig 3 indicated that about 1 μM AG-1031 effectively induced a loss of viability in all four lung carcinoma cell lines. Therefore, PC4 is essential for the viability of these tumor cells, and inhibition of PC4 activity by AG-1031 has potential as an alternative therapy of NSCLC. AG-1031 also had significant inhibitory effect on the viability of C6 cells that were derived from rat brain glioma [24, 25]. Furthermore, an analogue of AG-1031 (AG-1601) showed great inhibitory effect on the viability of human U251 glioma cells, but no such effect was observed on the mouse embryonic fibroblast NIH3T3 cells (S1 Fig).

Interestingly, western blot shows that the expression of PC4 is significantly reduced in A549 cells when the cells were treated with 0.3 μM of AG-1031 for 72 hours (Fig 3C).

### AG-1031 inhibited tumor growth in A549 cell xenografts

**Fig 4:**
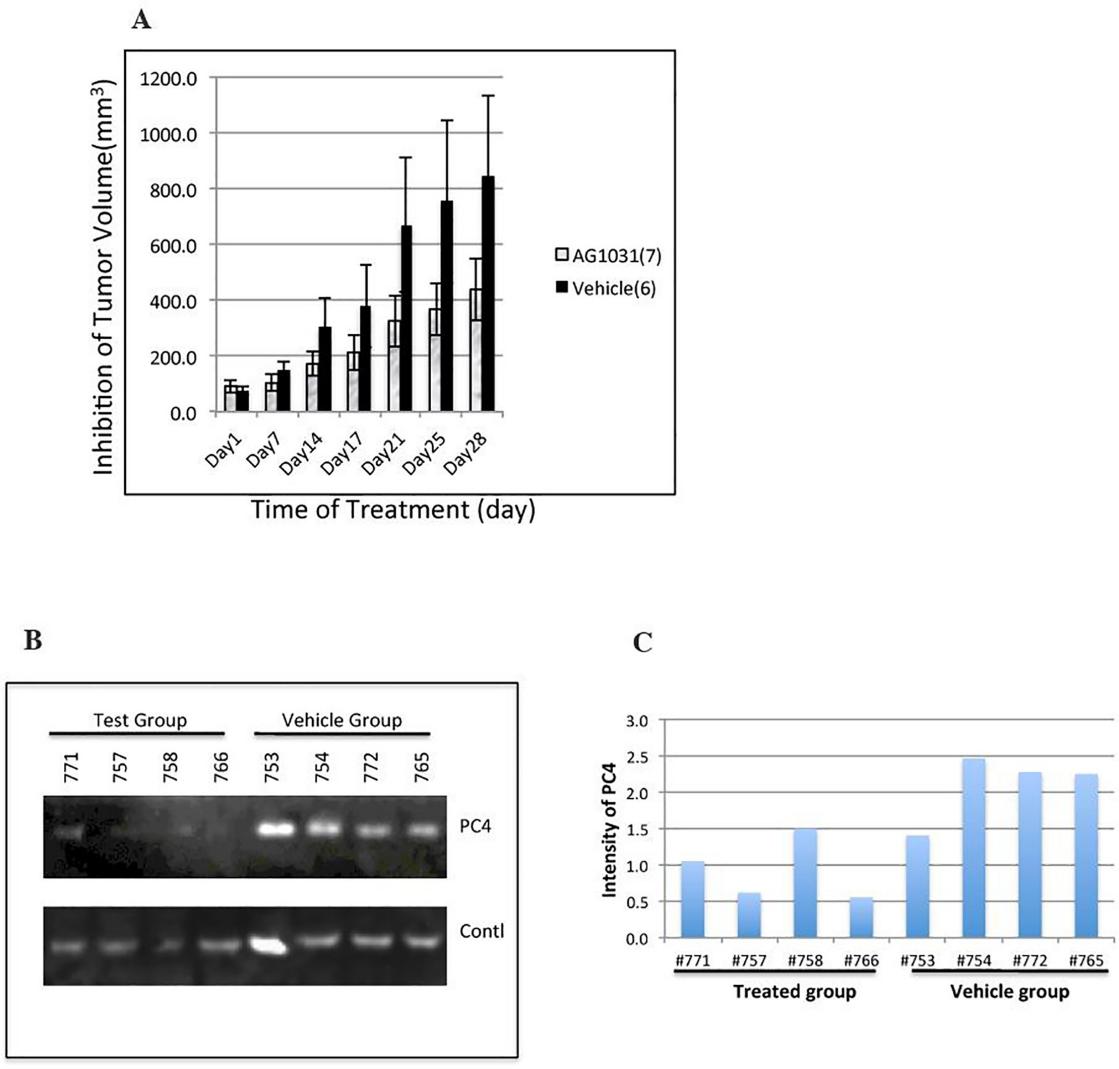
Efficacy of AG-1031 for A549 cell xenografts. A549 cell xenografts were produced in 7 mice and treated with AG-1031 (“AG-1031”) every other day. The tumor size was measured and compared to the control group of 6 mice without AG-1031 treatment (“vehicle”). Panel A compares the average tumor size between treated (“AG-1031”) and untreated (“vehicle”) groups. Western blot analysis revealed a decreased level of PC4 protein in most AG-1031-treated tumor tissues compared to untreated tumor tissues (panel B). Expression of TATA binding protein (TBP) was used as an internal control. The density of PC4 expression was measured and the relative inhibition was plotted (Fig 4C).

Xenografts were established by inoculating 8 weeks-old immunodeficiency mice (SHO) subcutaneously with NSCLC A549 cells. When tumors reached 75–90 mm^3^ in size, the Xenografts were randomly assigned either to an experimental group (AG-1031 treated) or a control group (untreated). Tumor-bearing mice in the experimental group were treated with AG-1031 (40 μg/mouse) in dIH2O via intraperitoneal (IP) injections every other day for 4 weeks. Mice in the control group were injected with dIH2O alone. Tumor size was measured with sliding calipers twice a week. As shown in Fig 4, AG-1031 inhibited tumor growth (p<0.05) in treated animals compared to untreated controls starting from day 7 (Fig 4A). On average, the tumor size of experimental group was 439 mm^3^ at day 28, where as the control group was 843 mm^3^. After normalizing for tumor size at day 1, the overall tumor size of treated group was 43% compared to the size of the control group. Western blot analysis revealed that the expression of PC4 in tumor tissue extract in most AG-1031 treated animals was much lower than that in untreated control animals (Fig 4B), suggesting that the reduced growth of tumors in A549 xenografts in mice treated with AG-1031 could result from a reduced level of PC4 protein.

The toxicity of AG-1031 was studied by monitoring the body weight of the tested animals. No dramatic change was observed between treated group and control group during the entire test period (S2 Fig). Furthermore, no sign of disease and no death were observed from AG-1031-treated animals.

**Fig 5:**
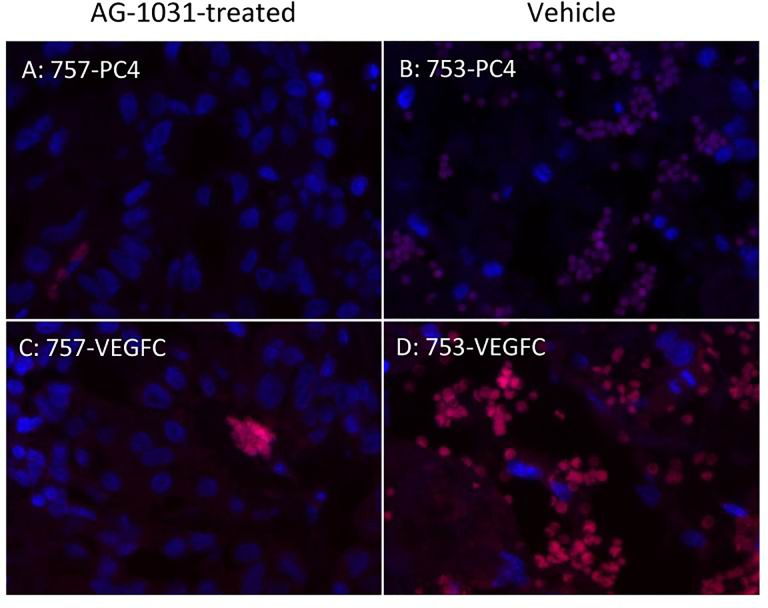
Down-regulation of PC4 correlates with down-regulation of VEGFC in AG-1031 treated tumor tissues. Tumor tissues from AG-1031-treated A549 xenograft (#757) and from untreated xenograft (#753) were stained either with monoclonal antibody against PC4 (A and B) or with polyclonal antibody against VEGFC (C and D). Primary antibodies were detected using IgG-conjugated with rhodamine (A and B), Alexa Fluor 568 (C and D) and nuclei with DAPI (A, B, C and D).

It was reported that down-regulation of PC4 by sequence-specific siRNA could induce apoptosis of NSCLC cells, which might result from the alteration of downstream factors, including PCNA, cyclin D1, p21, poly ADP-ribose polymerase (PARP), VEGFC, VEGFD and VEGFR3 [18, 22]. To determine whether or not inhibition of tumor growth by AG-1031 in A549 xenografts resulted from down-regulation of VEGFC as well as PC4, immunohistochemistry staining was used to correlate PC4 expression with VEGFC expression in tumor tissues treated with AG-1031 compared with tissues from untreated animals. A comparison of the effects of AG-1031 (Fig 5A and 5C) with the effects of the vehicle (Fig 5B and 5D) shows that the tumor tissue from AG-1031 treated animals had both a significantly reduced level of PC4 protein and a significantly reduced level of VEGFC, a well-studied key mediator of angiogenesis in cancer [22]. The same result was observed in the *in vitro* study with western blot analysis, in which both PC4 and VEGFC expression were significantly reduced in A549 cells when the cells were treated with 3μM concentration of AG-1031 for 72 hours.

### AG-1031 prevented tumor development in A549 cell tumor models

**Fig 6:**
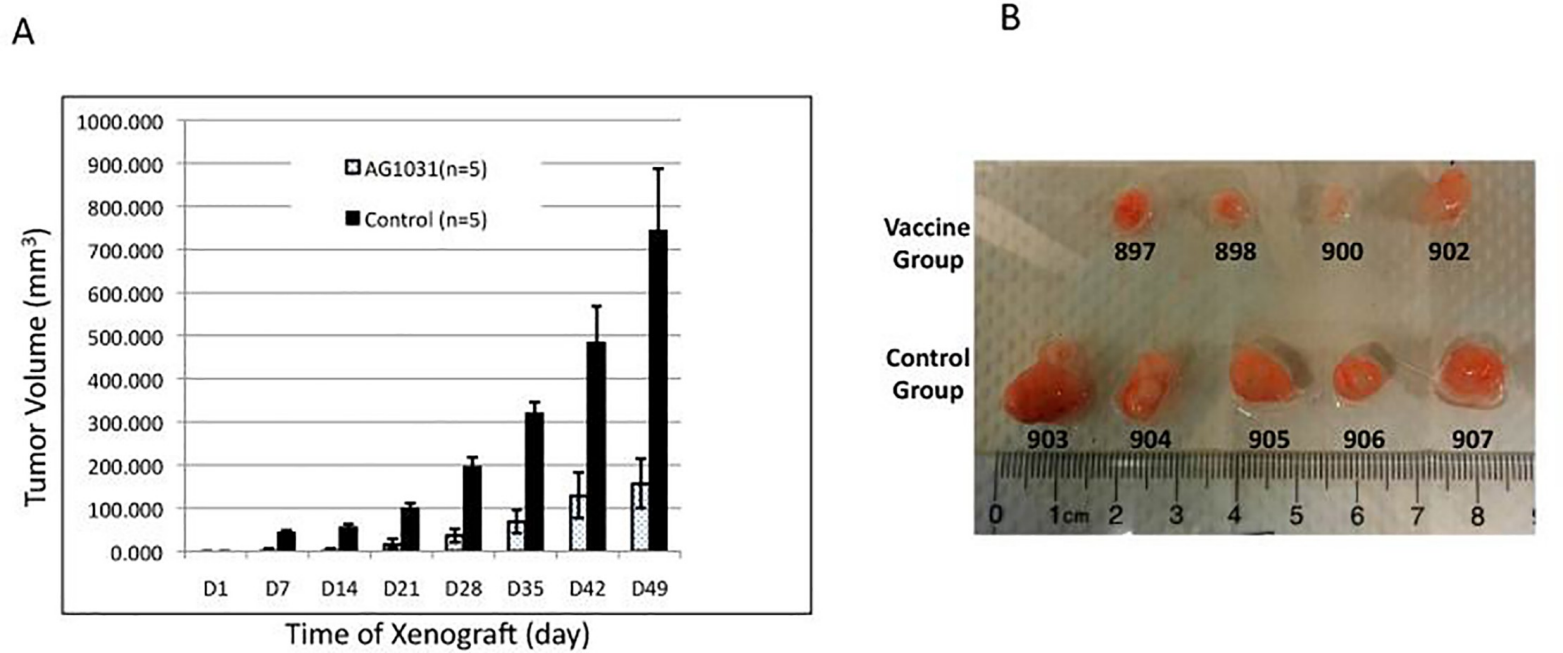
Pre-treatment with AG-1031 prevents tumor development. AG-1031 was pre-injected (IP) into mice once a week for 3 weeks before they were inoculated with A549 cells. (A) The tumor size of both treated (AG-1031) and untreated (Control) groups was measured and the average tumor size was compared. (B) Tumors from both groups were dissected on day 49, and individual tumors from each group are shown. Since one in five animals (#901) in the treated group failed to develop any tumor, even until day 49, only 4 tumors were shown in the treated group.

As a general transcription co-activator that is capable of mediating transcription activation of diverse genes, including those regulator genes that play essential roles in different stages of tumorigenesis, PC4 might act as an upstream inducer of tumor cells. Limitation of PC4 activity might therefore also prevent tumor development. To address this hypothesis, mice were pre-injected via IP with AG-1031 three times before inoculation with A549 cells. Tumor formation was monitored every 5 to 6 days. Surprisingly, tumors were not observed in any of the AG-1031 pre-treated mice by day 5 following inoculation with A549 cells, whereas all of the animals in the control group exhibited tumor development. Even by day 49, one of the five AG-1031 pre-treated animals did not develop tumors and the average tumor size in the remaining four animals was significantly smaller than the size in the control group (Fig 6, p<0.05). (Note: Day1 in this figure represent the day inoculating the tumor cells to the mice)

The transcription coactivator PC4 is a general cofactor mediating transcriptional activation of many class II and class III genes, including most oncogenes and genes encoding for other cancer-related proteins [18–20, 22]. Furthermore, PC4 is also a heterochromatin protein mediating chromatin organization by inducing chromatin condensation, suppressing spontaneous DNA damage and promoting genome stability by binding to single-stranded DNA, and activating double-stranded break repair by enhancing rejoining of double stranded breaks [11].

The relationship of PC4 with cancer has been widely studied in the last decade [17–22]. In addition to that high expression of PC4 has been characterized in different tumor tissues, siRNA knockdown of PC4 expression in human NSCLC cells and in NSCLC tumor models resulted in an inhibition of growth of tumor cells or tumors in xenografted animals [18, 22]. Further studies indicated that PC4 could induce the expression of VEGF-C, -D and VEGFR3 in angiogenesis of lung adenocarcinoma, suggesting that PC4 may function as an upstream inducer and mediate the expression of downstream factors necessary for angiogenesis at transcription level, such as VEGFs and VEGFRs [22]. PC4 is indeed one of the 46 common cancer signatures that were identified from large-scale microarray data integration [21]. Similar to histone deacetylase (HDAC) inhibitors, down-regulation of PC4 by siRNA also led to antitumorigenic effects on NSCLC cells including growth arrest, apoptosis and induction of differentiation [18]. Thus, PC4 could be a potential and important therapeutic target for cancer, including NSCLC.

Using a newly developed high-throughput screening platform, we were able to identify a single small molecule, AG-1031, that was approved for clinical use with a non-oncology indication. We found that AG-1031 preferentially inhibited PC4 dsDNA binding activity in vitro, an essential activity that correlates with its transcription activity. In vitro transcription studies indicated that AG-1031 could inhibit PC4-dependent transcription, but has less effect on PC4-independent transcription. Reduced activity of PC4 induced by AG-1031 led to the inhibition of proliferation of different NSCLC cell lines. Furthermore, injection of AG-1031 into NSCLC A549 cell xenografted mice resulted in a cytostatic inhibition of tumor growth. By analysis of protein contents of tumor tissues, we found that such an effect of inhibited tumor growth might be the consequence of reduced PC4 activity, subsequently resulting in a down-regulation of other downstream factors, such as VEGFC, that are activated by PC4. Surprisingly, pre-injection of AG-1031 into A549 xenografted animals revealed not only reduced tumor growth but also limited tumor development in an NSCLC tumor model. These observations demonstrate that PC4 could be an alternative therapeutic target and AG-1031 may represent a new class of therapeutics for NSCLC.

Development of cancer can occur through two main mechanisms [26, 5]. One is activation of oncogenes enhanced by either mutational activation or epigenetic activation. On the other hand, silencing (inactivation) of tumor suppressor genes by silencing mutations or by epigenetic inactivation represents the second mechanism of tumorigenesis [27]. Although the mechanism of AG-1031 action in preventing tumor formation is unknown, it is possible that PC4, a non-histone chromatin binding protein might, in addition to its direct roles as a cofactor in transcription and other processes, act as an oncogenic factor and contribute to initiating development of cancer. Therefore, limiting of PC4 activity or lower PC4 expression level by PC4 inhibitors may have the potential to prevent early stage development of cancer, which has been a longstanding goal of cancer research. Development of NSCLC vaccines have been initiated with different targeted antigens, including MAGE-A3, MUC1, TGF-beta2, EGF and IL2, among other markers, but the clinical results have been disappointing [26]. Since PC4 is a nuclear protein that would be unlikely to be targeted by an antibody, AG-1031 may thus be a potentially effective preventive drug for cancer, at least for NSCLC where it could supplement the indicated NSCLC therapy.

Indicated modalities for NSCLC, including tyrosine kinase inhibitors and monoclonal antibodies that target either EGFR, ALK or PD1, have been demonstrated to improve clinical outcome in target-positive tumors. In patients with target-negative tumors, however, most of these drugs have showed very limited impact [2]. In the present study, we have shown efficacy of AG-1031 in the treatment of NSCLC in animal models and it remains of interest to further explore if we can offer a combinational therapy with above targeted drugs, including tyrosine inhibitors and monoclonal antibodies, to maximize the efficacy for NSCLC treatment.

Based on the study of structure-activity relationship (SAR) of AG-1031, we have selected a group of commercially available analogues and a group of newly synthesized analogues for their activity to inhibit PC4-dsDNA binding in vitro. A compound, AG-1503, was identified from commercially available analogues. Although its inhibitory effect on the proliferation of NSCLC cell lines was not as good as AG-1031, it showed, however, a better inhibitory activity on the proliferation of C6 rat glioma cell line [25]. An additional compound, AG-1601, was identified from newly synthesized analogues. It showed much better inhibitory effect on PC4-dependent transcription and proliferation of both NSCLC and glioma cell lines. These observations have enriched our pipelines of new strategy for the treatment and prevention of NSCLC, and probably other cancers as well.

## Conclusions

Use of a high-throughput screen assay, we were able to identify a small molecule of PC4 inhibitor, AG-1031, that was originally approved by the U.S. Food and Drug Administration for non-oncology indication. AG-1031 inhibited PC4-dependent transcription in vitro in a concentration-dependent manner. Both in vitro and in vivo studies indicated that AG-1031 inhibited proliferation of non-small cell lung cancer derived cell lines and the growth of tumors formed from A549 cell xenograft animals. Furthermore, pre-injection of AG-1031 in xenograft animals not only reduced tumor size, but also limited tumor formation in 20% of the animals. As PC4 activity is regulated by casein kinase II phosphorylation and only the dephosphorylated form of PC4 is functionally active [13–15], the reduced levels of PC4 detected from AG-1031-treated cell lines or animal tissues correspond to the size of unphosphorylated form, We further conclude that PC4 plays an important role in pathogenesis of non-small cell lung cancer and other types of tumors. Therefore, AG-1031 and other PC4 inhibitors might represent an alternative strategy for cancer therapy.

## Supporting information

S1 Fig AG-1031 analogue AG-1601 showed great inhibitory effect on the viability of U251 cells that were derived from human glioblastoma, but not such an effect was observed on the NIH3T3 cells that were derived from mouse embryo. (DOCX)

S2 Fig AG-1031 does not affect the animal’s body weight and survival. The body weights of individual mice were measured and recorded during the experimental period. No significant difference was observed between the animals treated with AG-1031 (AG-1031) and animals of control group (Vehicle).
(DOCX)

S1 Raw images (TIF)
