# Supplementary figures and images for: Efficacy of a small molecule inhibitor of the transcriptional cofactor PC4 in prevention and treatment of non-small cell lung cancer
Source: PLoS One. 2020 Mar 31;15(3):e0230670. doi: 10.1371/journal.pone.0230670 (PMC7108703; doi:10.1371/journal.pone.0230670)

S2 Fig


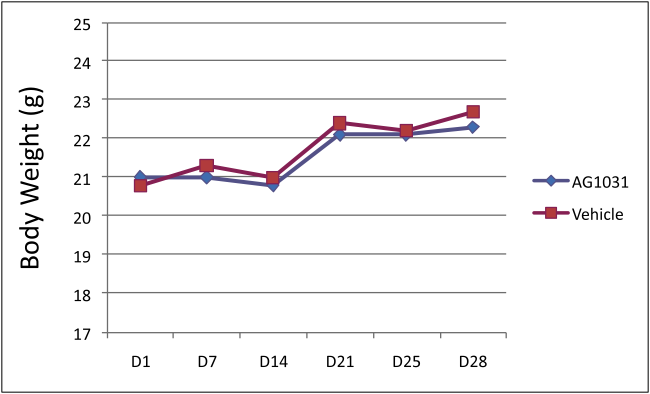

Supplement: S2 Fig — The body weights of individual mice were measured and recorded during the experimental period. No significant difference was observed between the animals treated with AG-1031 (AG-1031) and animals of control group (Vehicle). (DOCX) [file pone.0230670.s002.docx]

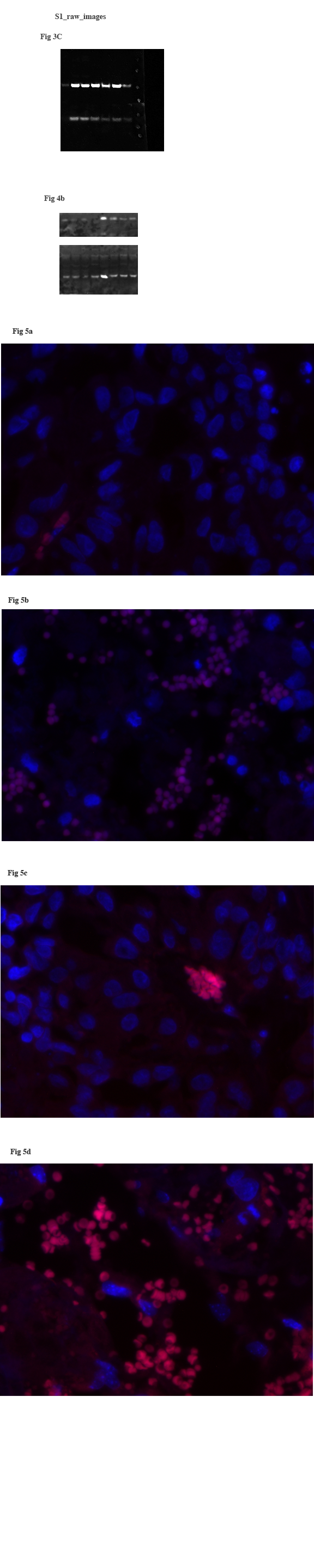

Supplement: S1 Raw images — (TIF) [file pone.0230670.s003.tif]
